# A Single Amino Acid Substitution in Elongation Factor G Can Confer Low-Level Gentamicin Resistance in *Neisseria gonorrhoeae*

**DOI:** 10.1128/aac.00251-22

**Published:** 2022-04-25

**Authors:** Concerta L. Holley, Vijaya Dhulipala, Jacqueline T. Balthazar, Adriana Le Van, Afrin A. Begum, Shao-Chun Chen, Timothy D. Read, Mitch Matoga, Irving F. Hoffman, Daniel Golparian, Magnus Unemo, Ann E. Jerse, William M. Shafer

**Affiliations:** a Department of Microbiology and Immunology, Emory University School of Medicine, Atlanta, Georgia, USA; b Department of Microbiology and Immunology, Uniformed Services University, Bethesda, Maryland, USA; c Institute of Dermatology and Hospital for Skin Diseases, Chinese Academy of Medical Sciences and Peking Union Medical College, Nanjing, People’s Republic of China; d National Center for Sexually Transmitted Diseases Control, Chinese Center for Disease Control and Prevention, Nanjing, People’s Republic of China; e Division of Infectious Diseases, Department of Medicine, Emory University School of Medicine, Atlanta, Georgia, USA; f The Emory Antibiotic Resistance Center, Emory University School of Medicine, Atlanta, Georgia, USA; g UNC Project Malawi, Lilongwe, Malawi; h Department of Medicine, University of North Carolina at Chapel Hill, Chapel Hill, North Carolina, USA; i WHO Collaborating Centre for Gonorrhoea and Other STIs, National Reference Laboratory for STIs, Department of Laboratory Medicine, Clinical Microbiology, Faculty of Medicine and Health, Örebro University, Örebro, Sweden; j Institute for Global Health, University College London, London, United Kingdom; k Laboratories of Bacterial Pathogenesis, Veterans Affairs Medical Center, Decatur, Georgia, USA

**Keywords:** *Neisseria gonorrhoeae*, gentamicin, resistance, *fusA*, spontaneous mutant

## Abstract

The continued emergence of Neisseria gonorrhoeae isolates which are resistant to first-line antibiotics has reinvigorated interest in alternative therapies such as expanded use of gentamicin (Gen). We hypothesized that expanded use of Gen promotes emergence of gonococci with clinical resistance to this aminoglycoside. To understand how decreased susceptibility of gonococci to Gen might develop, we selected spontaneous low-level Gen-resistant (Gen^R^) mutants (Gen MIC = 32 μg/mL) of the Gen-susceptible strain FA19. Consequently, we identified a novel missense mutation in *fusA*, which encodes elongation factor G (EF-G), causing an alanine (A) to valine (V) substitution at amino acid position 563 in domain IV of EF-G; the mutant allele was termed *fusA2*. Transformation analysis showed that *fusA2* could increase the Gen MIC by 4-fold. While possession of *fusA2* did not impair either *in vitro* gonococcal growth or protein synthesis, it did result in a fitness defect during experimental infection of the lower genital tract in female mice. Through bioinformatic analysis of whole-genome sequences of 10,634 international gonococcal clinical isolates, other *fusA* alleles were frequently detected, but genetic studies revealed that they could not decrease Gen susceptibility in a similar manner to *fusA2*. In contrast to these diverse international *fusA* alleles, the *fusA2-*encoded A563V substitution was detected in only a single gonococcal clinical isolate. We hypothesize that the rare occurrence of *fusA2* in N. gonorrhoeae clinical isolates is likely due to a fitness cost during infection, but compensatory mutations which alleviate this fitness cost could emerge and promote Gen^R^ in global strains.

## INTRODUCTION

Gonorrhea is the second most commonly reported bacterial sexually transmitted infection in the United States (1), and there are an estimated 87 million gonorrhea cases among adults globally per year (2). For over 80 years, antibiotic therapies have been the mainstay of curing gonorrheal infections and reducing the spread of infection in the community. However, one consistent pattern during this period has been the continued emergence of Neisseria gonorrhoeae (Ng) strains which express clinical resistance to all introduced antibiotics (3), and multidrug-resistant (MDR) isolates have been reported globally (4–6). For these reasons, Ng is considered an urgent health threat pathogen by both the Centers for Disease Control (CDC) and the World Health Organization (WHO) (7–9). Ceftriaxone (Cro), used in combination with azithromycin (Azm) (10–13) or in high-dose monotherapy (14–17), is the final drug for empirical first-line treatment of gonorrhea.

The emergence of MDR Ng clinical isolates has prompted the development and clinical testing of new antibiotics, the use of molecular diagnostics to identify isolates susceptible to previously used antibiotics (e.g., ciprofloxacin), and the use of existing antibiotics in new gonorrheal treatment regimens (6, 18, 19). With respect to existing antibiotics which have been used to treat other bacterial infections, gentamicin (Gen) is known to have anti-gonococcal action and has been used in many countries when recommended first-line gonorrheal therapy fails (6) and in Malawi as front-line empirical gonorrheal therapy, i.e., in combination with doxycycline (20, 21). However, there are currently no interpretative criteria for Gen MICs set by the Clinical and Laboratory Standards Institute (CLSI) or the European Committee on Antimicrobial Susceptibility Testing (EUCAST). MICs of ≥32 μg/mL have been used by the World Health Organization and in Malawi as the Gen resistance breakpoint for gonococci. In Malawi, Gen has been routinely used to treat suspected gonococcal infections for over 25 years, and isolates have mainly been uniformly susceptible to Gen *in vitro* (20); Gen treatment failures have been verified, likely due to pharmacokinetic/pharmacodynamic reasons (21). Similarly, a study of 1,366 gonococcal isolates from 17 European countries showed that most isolates were Gen-susceptible (22). In China, gonococcal isolates have also demonstrated full Gen susceptibility (23). Of note, a recent Gonococcal Isolate Surveillance Project (GISP) study suggested that Gen susceptibility might be decreasing even though all isolates included in the study were considered susceptible, with MICs of ≤8 μg/mL (24).

If Gen use becomes more prevalent in the USA and elsewhere for gonorrhea treatment, it is critical to understand how Ng might develop clinical resistance. In this respect, high-level (MIC ≥ 256 μg/mL) aminoglycoside resistance in other bacteria can be due to rRNA modifications based on nucleoside changes or to the action of RNA methylases that impair antibiotic binding (25, 26). Additionally, a lower level of aminoglycoside resistance, which may have clinical significance for efficacious treatment of infections, can be mediated by multidrug efflux pumps, mutations that impact membrane integrity or homeostasis, or mutations within the *fusA* gene, which encodes elongation factor G (EF-G) (27–33). To date, clinical Ng isolates expressing either level of Gen resistance (Gen^R^) have not been reported. With respect to the possibility of low-level Gen^R^ development, most Ng isolates remain highly susceptible (MICs of <16 μg/mL), but a small percentage (2%) of USA isolates have a reduced susceptibility (MICs of 16 μg/mL) (24). In this study, we sought to examine how low-level Gen^R^ may develop *in vitro* and report that a single amino acid substitution in EF-G can confer low-level Gen^R^.

## RESULTS

### Identification of a *fusA* mutation associated with low-level Gen^R^ in Ng.

A recent study suggested that the use of Gen for gonococcal infections would likely lead to emergence of resistance, although the potential mechanisms for this were not discussed (34). To understand how low-level Gen^R^ may arise, in two separate experiments, we selected and screened (see Materials and Methods) for spontaneous mutants of the Gen-susceptible N. gonorrhoeae strain FA19 (Gen MIC = 8 μg/mL) which could grow on gonococcal base (GCB) agar containing 1× Gen MIC; these colonies arose at frequencies ranging from 1.5 × 10^−8^ to 5.2 × 10^−7^. Randomly-picked colonies from the selective agar were reanalyzed for levels of susceptibility to Gen and other antibiotics, including aminoglycosides, using an agar dilution assay. From this analysis, we identified a clone that expressed a 4-fold decrease in Gen susceptibility (MIC = 32 μg/mL) (Table 1) compared to the parental strain FA19. Additionally, compared to FA19, this spontaneous mutant also exhibited a 4-fold decrease in susceptibility to tobramycin (Tob) and a 2-fold decrease in susceptibility to kanamycin (Kan), but was equally susceptible to erythromycin (Ery), polymyxin B (Pmb), spectinomycin (Spt), and streptomycin (Str) (Table 1). Because the MIC of Gen against this mutant was at the previously suggested breakpoint (20, 21), this phenotype was termed low-level Gen^R^.

**TABLE 1:** Antimicrobial susceptibility of the parent strains and Gen^R^ mutants^*a*^

Strain	Antimicrobial class MICs (μg/mL)							
	Aminoglycosides				Aminocyclitol	Macrolides		Cationic peptide
	Gen	Tob	Kan	Str	Spt	Ery	Azm	Pmb
FA19	8	8	32	16	32	0.25	0.125	100
FA19 Gen^R^	32	32	64	16	32	0.25	0.125	100
FA19 *fusA2*	32	16	64	16	32	0.25	0.125	100
WHO X	16	8	32	16	32	2	1	400
WHO X *fusA2*	32	16	64	16	32	2	1	400

a All MIC values are representative results from 3 to 5 independent determinations. Gen, gentamicin; Tob, tobramycin; Kan, kanamycin; Str, streptomycin; Ery, erythromycin; Azm, azithromycin; Spt, spectinomycin; Pmb, polymyxin B.

In order to determine the potential mechanism(s) for the Gen^R^ phenotype of the spontaneous mutant, we performed whole-genome sequencing (WGS) using genomic DNA from FA19 and the spontaneous mutant. Comparative bioinformatic analysis revealed a total of 41 nonsynonymous single nucleotide mutations (SNPs) and 1 indel in intragenic sequences of the mutant (Table S1 in the supplemental material). Interestingly, one missense mutation (A563V) was identified in the protein-coding sequence of *fusA*, which encodes elongation factor G (EF-G). We termed this *fusA* allele *fusA2.*

**FIG 1:**
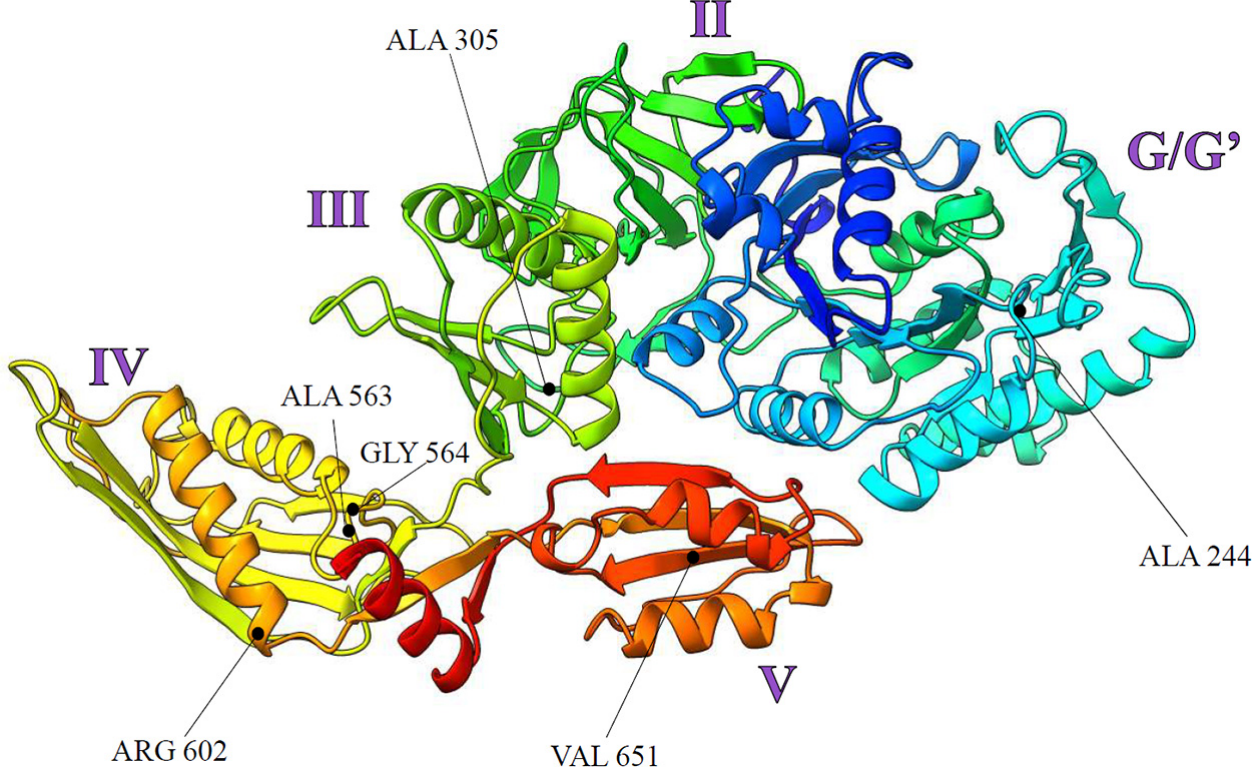
The predicted structure of the gonococcal elongation factor G (EF-G) protein. Three-dimensional ribbon structure of *fusA-*encoded EF-G protein from FA19. Protein was predicted by I-TASSER (https://zhanglab.ccmb.med.umich.edu/I-TASSER/) using the predicted amino acid sequence (WP_003690097.1) and visualized by Chimera (https://www.rbvi.ucsf.edu/chimera). EF-G domains were predicted using the Pfam database (https://pfam.xfam.org/) and are indicated in purple. Locations of key amino acids included in this study are labeled in black.

The *fusA2* allele was of interest because earlier work showed that mutations in E. coli *fusA* could decrease bacterial susceptibility to Gen and other aminoglycosides (26). The missense mutation in *fusA2* would cause an alanine (A) to valine (V) change at amino acid position 563 (A563V) in the gonococcal EF-G protein. The Ng *fusA* gene resides within the ribosomal gene cluster which contains the essential *rpsG* and *rpsL* genes (35). Ng EF-G is a 701-amino acid protein with a predicted weight of 77.18 kDa and an isoelectric point of 4.99. Ng EF-G has 74.25% homology to the E. coli EF-G amino acid sequence, with a classical elongation factor protein structure consisting of five domains: globular I, II, III, IV, and V (Fig. 1). In E. coli, the globular I domain interacts directly with domains II and V and binds to the ribosome. Domain III is likely involved in the activation of GTP hydrolysis, while domain IV is critical for translocation. Domain IV, wherein A563 resides, protrudes from the molecule, and its movement toward the A site of the 30S ribosomal subunit helps promote the translocation of peptidyl tRNA from the A to the P site (36). Notably, A563 is conserved across EF-G homologs in E. coli, N. meningitidis, and P. aeruginosa (Fig. S1 in the supplemental material), suggesting that it may have an important role in EF-G protein function.

### Genetic linkage of *fusA2* to low-level Gen^R^.

To confirm that the A563V mutation encoded by *fusA2* could confer low-level Gen^R^, we amplified the *fusA2* allele from the spontaneous FA19 Gen^R^ mutant and cloned it into pBad-Topo. We used this plasmid to transform parent strain FA19 for increased Gen resistance; because pBAD does not replicate in Ng, the wild-type (WT) chromosomal *fusA* gene would be replaced by *fusA2* by allelic exchange. Gen^R^ transformants were obtained, and the representative colonies mostly phenocopied the antibiotic susceptibility profile of the spontaneous mutant in that the *fusA2* transformant displayed a 4-fold increase in Gen resistance, but only a 2-fold increase in Tob resistance; the MIC values for antibiotics against the representative transformant are shown in Table 1. The presence of the single missense mutation at *fusA* codon 563 in the representative transformant was confirmed by DNA sequencing of a PCR product which encompassed the entire gene (data not shown). We recognize that it is possible that mutations outside *fusA* are required to achieve the level of Tob resistance observed in the spontaneous Gen^R^ mutant.

In order to determine if possession of *fusA2* in a MDR Ng strain would also cause low-level Gen^R^, we used strain WHO X (previously termed H041 [37]) in transformation experiments; the Gen MIC against WHO X was 16 μg/mL. Using pBad-*fusA2* as the donor DNA, we obtained WHO X transformants which had a 2-fold increase in the Gen MIC compared to that of the parental strain (Table 1). The WGS of a representative WHO X transformant confirmed the presence of *fusA2* and this was designated WHO X *fusA2* (Table S2). We noted that WHO X and WHO X *fusA2* also differed by a single SNP in one of four rRNA-coding genes, but this SNP was not apparent in the WGS obtained for FA19 and its spontaneous mutant, suggesting that it is not required for the low-level Gen^R^ phenotype linked to *fusA2*. Further, a comparison of intra- and intergenic SNPs and indels of the FA19 *fusA2* and WHO X *fusA2* strains revealed that only the *fusA* SNP (A563V mutation) was common. Thus, based on the collective genetic and DNA sequencing results, we concluded that the presence of the A563V substitution in EF-G was the reason for the low-level Gen^R^ phenotype in *fusA2*-bearing Ng.

### *fusA2* does not impact gonococcal growth or protein synthesis.

Possession of *fusA2* did not impact the growth rate of gonococci in GCB broth (Fig. S2A). To assess whether *fusA2* influences the fidelity of protein synthesis, we assessed [^3^H]l-leucine incorporation during growth of FA19 and FA19 *fusA2* in GCB broth. As is shown in Fig. S2B, strains FA19 and FA19 *fusA2* did not differ in [^3^H]l-leucine incorporation, suggesting that possession of *fusA2* does not significantly impact protein synthesis, which is consistent with the absence of a growth defect. We also considered the possibility that, in the presence of Gen, protein synthesis inhibition would be impacted by *fusA2*. To test this possibility, we examined radiolabeled l-leucine incorporation by strains FA19 and FA19 *fusA2* in the presence of 0.25× or 1× the Gen strain MIC. We found that, in the presence of Gen, there was no significant difference in l-leucine incorporation between the *fusA* WT and *fusA2* isogenic strains (Fig. S2B).

### *fusA* alleles in international gonococcal isolates.

Given that genetic studies linked the Gen^R^ phenotype of the FA19 and WHO X transformant strains to *fusA2*, we sought to determine if this allele is present in international Ng clinical isolates. In this respect, an initial investigation was performed on 58 clinical isolates which were part of an earlier study that tested the phylogenetic relationships of isolates bearing antibiotic-resistance determinants (38). Although the *fusA* sequence was highly conserved (>99% identity to the FA1090 sequence), we found 2 missense mutations (A305P/T, V651F) and 1 nonsense mutation (insertion after K683) that would lead to early translational termination in this cohort of isolates, which would result in an EF-G protein of 691 amino acids. Based on this initial analysis, we expanded the search for *fusA2* to 654 publicly available genomes in the NCBI database (https://www.ncbi.nlm.nih.gov/genome/864). This analysis found 2 additional missense *fusA* mutations in clinical isolates (G91R and R602H), but did not identify the A563V mutation. Given that our initial meta-analysis did not identify the *fusA2*-encoded mutation (A563V) in EF-G, we expanded our analysis to include all the gonococcal genomes present in the PubMLST database (https://pubmlst.org/organisms/neisseria-spp) (39). Analysis of the 10,561 genomes contained in the database identified 51 additional missense mutations and 1 nonsense mutation that spanned the entirety of EF-G; a full list of these mutations and their continents of origin is presented in Table S3. Interestingly, we were able to identify the *fusA2*-encoded A563V mutation in one strain isolated from a patient in Saudi Arabia (isolate no. 989000093). In addition to this large collection of international N. gonorrhoeae isolates, we also performed DNA sequencing on a complete *fusA* PCR product of gDNA using the oligonucleotide primers FusA2 and FusA3 (Table S4) from 359 clinical isolates from China which were obtained in 2016; however, none of these were found to contain the *fusA2* allele. Lastly, bioinformatic analysis of WGS from 73 gonococcal isolates obtained from Malawi, where Gen is used in combination with doxycycline as a front-line empirical therapy for gonorrhea, did not reveal the presence of the EF-G A563V variant.

To test whether the previously-described *fusA* mutations could impart Gen^R^ in a similar manner to the *fusA2*-encoded A563V substitution, we performed site-directed mutagenesis on the FA19 *fusA* WT gene to introduce selected mutations (A244V, A305T, G564D, R602H, and V651F) which span EF-G into strain FA19 by transformation. For this purpose, we used pCR4 plasmid constructs in transformation experiments with selection using Gen at 12 μg/mL. We found that, of the plasmid constructs, only those encoding the EF-G G564D and V651F amino acid substitutions could transform FA19 for decreased Gen susceptibility (Table 2); DNA-sequencing of *fusA* gene PCR products in these transformants confirmed the presence of the individual mutation (data not shown). However, the Gen MIC of these transformants was only 16 μg/mL. Interestingly, rare colonies which appeared on selective agar in transformation experiments using plasmids bearing the A244V, A305T, or R602H mutations were subsequently found by DNA sequencing of PCR products to possess the *fusA2*-encoded A563V mutation (data not shown). (The occurrence of these spontaneous *fusA2* mutants emphasizes the relative ease with which *fusA2* can arise under laboratory conditions). To specifically construct transformants of FA19 with these *fusA* mutations, we transformed FA19 with dual plasmids: one with the *rpsL* allele which encodes Str resistance (Str^R^), and the other with a single site-directed *fusA* mutation (A244V, A305T, or R602H). We reasoned that, by congression (i.e., the simultaneous entry of two DNA molecules into a single recipient), a percentage of Str^R^ transformants would also have the *fusA* mutation at the WT chromosomal site. Using this strategy, we were able to identify Str^R^ transformants which also contained the desired *fusA* mutation, which was determined by DNA sequencing of PCR products which spanned *fusA*. Critically, unlike the *fusA2*-encoded A563V amino change, none of the site-directed mutations at codons A244, A305, and R602 could decrease Gen susceptibility in the host strain FA19 (Table 2).

**TABLE 2:** *fusA* mutations in gonococci and susceptibility to antimicrobials^*a*^

Predicted domain	*fusA* mutation	Antimicrobial class MICs (μg/mL)				
		Aminoglycoside		Macrolide	Cationic peptide	Fusidane
		Gen	Tob	Azm	Pmb	FA
	WT	8	8	0.25	100	0.0625
G/G’	A244V	8	8	0.25	100	0.0625
II	A305T	8	8	0.25	100	0.125
IV	A563V	32	16	0.25	100	0.0625
	G564D	16	16	0.25	100	0.0625
	R602H	8	8	0.25	100	0.0625
V	V651F	16	16	0.25	100	0.125

a All MIC values are representative results from at least 5 independent determinations. Gen, gentamicin; WT, wild-type; Tob, tobramycin; Azm, azithromycin; Pmb, polymixin B; FA, fusidic acid; G/G’, common name for Domain I which hydrolyzes GTP.

### Possession of *fusA2* imposes a fitness cost during experimental competitive infection of female mice.

**FIG 2:**
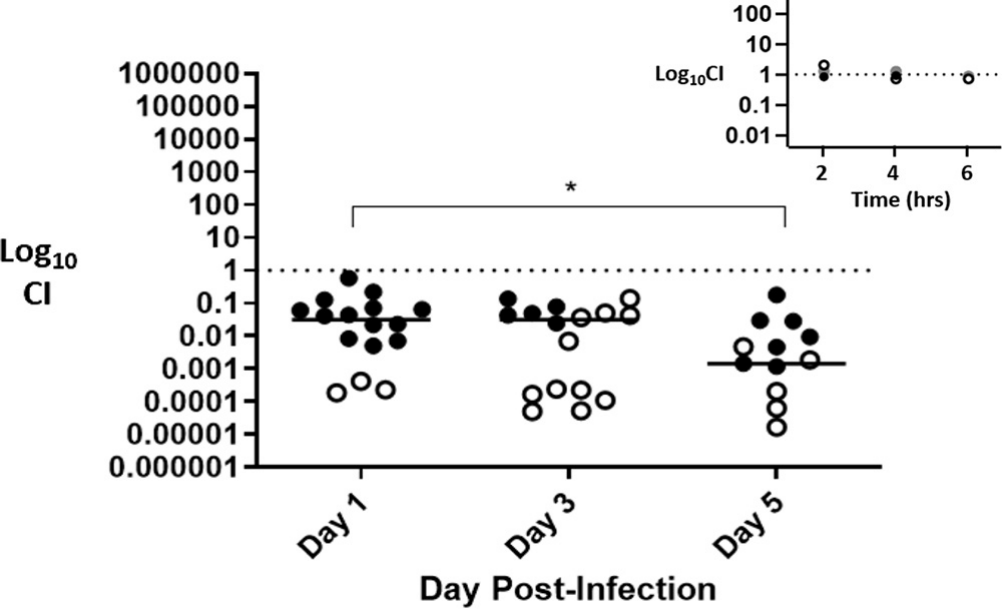
The *fusA2* mutant is attenuated relative to the parent strain during experimental genital tract infection in female mice. Estradiol-treated, female BALB/c mice were vaginally inoculated with a mixed suspension containing similar numbers of wild-type strain FA19 *rpsL* or FA19 *rpsL fusA2.* Vaginal swabs were quantitatively cultured on selective media to determine CFU counts for the parent strain and *fusA2* on days 1, 3, and 5 of infection, as described in Materials and Methods. The competitive index (CI) [*fusA2* CFU/parent CFU (output) divided by *fusA2* CFU/parent CFU (input)] was determined, and the log_10_ CI was plotted for each mouse. The *fusA2* strain showed a 50- to 100,000-fold decrease in fitness relative to the parent strain, with attenuation being greater on day 5 compared to day 1 (*P* < 0.04). Results are combined data from two experiments (*n* = 8 mice/experiment); open circles indicate that only the wild-type strain was recovered from vaginal swabs at the indicated time point. Horizontal bars represent the geometric means of the CI values. Results from three independent *in vitro* competition experiments in which FA19 *rpsL* and FA19 *rpsL*, *fusA2* were cocultured in supplemented GC broth are shown in the insert, with growth monitored by optical density. Dilutions of the mixed culture were plated onto gonococcal base (GCB) agar with streptomycin (Str, 100 μg/mL), with or without 8 μg/mL of gentamicin (Gen) to distinguish the two strains based on growth of only the *fusA2*-bearing strain on GCB agar + Gen and Str. No fitness differences were found during the early log (2 h), mid-log (4 h), or stationary (6 h) phases of growth (geometric mean CI = 1.2, 0.95, and 0.8 at each time point, respectively).

The infrequent occurrence of the *fusA2*-encoded A563V mutation in international clinical isolates may suggest that strains bearing this mutation are at a competitive disadvantage in the community due to a fitness cost during infection. To test this hypothesis, we conducted competitive infections in a female mouse model of Ng lower genital tract infection which we have used extensively to ascertain the fitness costs imposed by mutations which influence gonococcal virulence (40, 41). In duplicate experiments, we vaginally inoculated female BALB/c mice with a mixture containing similar numbers of FA19 *rpsL* and FA19 *rpsL fusA2* CFU; analysis of their growth during coculture in GCB broth revealed that neither strain had a competitive advantage at any growth phase (see insert in Fig. 2). Vaginal swabs were cultured on days 1, 3, and 5 postinfection and plated onto GCB agar with or without Gen at 8 μg/mL to allow distinction of the two strains. As shown in Fig. 2, FA19 *rpsL* significantly outcompeted FA19 *rpsL fusA2* over the course of the infection, suggesting that possession of *fusA2* imposed a fitness cost.

## DISCUSSION

As gonorrheal treatment options have continued to dwindle due to the resistance of Ng to current and past front-line antibiotics, the use of Gen to treat gonorrheal infections may become more prevalent. This study was undertaken to identify potential resistance determinants which could arise. Presently, the prevalence of Gen susceptibility depends largely on the region of study. In Malawi, China, Europe, and the U.S., Ng isolates showed universal susceptibility to Gen (MICs of ≤8 μg/mL) (21–24, 42). In contrast, intermediate or reduced susceptibility to Gen has been seen in Jamaica, India, South Africa, and Côte d’Ivoire (43–46). In relation to the significance of our study, both the South Africa and Côte d’Ivoire studies reported strains for which the Gen MIC values were as high as 32 μg/mL (45, 46).

Our finding of low-level Gen resistance in gonococci through a single amino acid substitution at position 563 in EF-G in a highly antimicrobial-susceptible strain (FA19) and a well-studied MDR-resistant strain (WHO X) demonstrates that Ng has the capacity to spontaneously develop low-level Gen resistance (MIC = 32 μg/mL) which may be of clinical significance. Although EF-G is an essential component of translational machinery, nonlethal mutations within the coding gene *fusA* have been reported as mechanisms of aminoglycoside resistance in other bacteria; consistent with the essential nature of EF-G, we have been unable to construct a *fusA* null mutant in the Ng strain FA19 (data not shown). For instance, an evolutionary study of Kan^R^ demonstrated that increasing levels of resistance in E. coli began with a single point mutation in *fusA* (32). Additionally, both P. aeruginosa and A. baumannii evolved *fusA* mutations after repeated exposure to Tob (31). In that study, it was noted that at least one *fusA* mutation was found in every Tob-treated lineage. At present, we do not understand the mechanism by which the A563V substitution in gonococcal EF-G confers low-level aminoglycoside resistance in gonococci. Although it is located in domain IV, which promotes the translocation of peptidyl tRNA from the A to the P site in ribosomes (36), the mutation studied herein does not detectably impact gonococcal protein synthesis or Gen inhibition of protein synthesis compared to the *fusA* WT gene. Other groups have proposed that mutations which change EF-G structure may prevent or loosen binding of aminoglycosides to helices H44 or H69, which would lower drug affinity for the ribosome, increasing the MIC (33). Mutations in domains IV and V may dynamically alter the functionality of the protein. Alternatively, it may be that *fusA2* has secondary effects on the expression of other intrinsic aminoglycoside resistance systems, and this will be the subject of future investigations.

It is noteworthy that the *fusA2*-associated A563V mutation is exceedingly rare in international gonococcal isolates. While the mutation does not cause an *in vitro* growth defect in laboratory media, it does impose a significant fitness cost during experimental competitive infection of the lower genital tract in female mice (Fig. 2). This fitness cost may explain why this allele is rarely seen in international clinical isolates (Table S3); the mechanism by which it causes this fitness cost in female mice is unclear and the subject of ongoing studies. However, we stress that if Gen usage for treating gonorrheal infection becomes more prominent worldwide, compensatory mutations which relieve the fitness cost without changing Gen^R^ may develop, allowing the proliferation of *fusA2*-bearing strains in communities. Furthermore, based on our studies and those of others (21–24, 42–46), it will also become critical to include Gen in routine antimicrobial susceptibility testing so that Ng isolates with decreased susceptibility to this aminoglycoside can be identified and studied.

## MATERIALS AND METHODS

### Bacterial strains, plasmids, and primers.

Ng strains FA19 and WHO X and their isogenic genetic derivative strains, along with the plasmids used and their E. coli hosts, are listed in Table S5. The oligonucleotide primers used in this study are listed in Table S4. E. coli strains were routinely cultured on Luria-Bertani (LB) agar or in LB broth (Difco, Sparks, MD) containing 50 μg/mL Kan, 100 μg/mL ampicillin, or 100 μg/mL chloramphenicol as necessary. Gonococci were grown on gonococcal base agar (Difco, Sparks, MD) containing Kellogg’s supplements I and II at 37°C under 5.0% (vol/vol) CO_2_ (47). Liquid cultures of gonococci for growth assays were begun by inoculating plate-grown cells in pre-warmed GCB broth containing Kellogg’s supplements I and II and 0.043% (wt/vol) sodium bicarbonate and growing them in a 37°C water bath with shaking.

### MIC determination.

The MICs of antimicrobials were determined by the agar dilution method (48). Antimicrobials were purchased from Sigma Chemical Co. (St. Louis, MO). Plates were incubated for 48 h at 37°C under 5.0% (vol/vol) CO_2_-enriched atmosphere before being photographed for data storage purposes.

### Isolation of spontaneous Gen^R^ mutants.

Spontaneous Gen^R^ mutants were isolated as in a previous study (49), with modifications. Briefly, FA19 was exposed to sublethal Gen concentrations (0.2 μg/mL) on GCB agar containing Kellogg’s supplements I and II at 37°C under 5.0% (vol/vol) CO_2_ overnight. The biomass was collected and spread on GCB plus Gen at 4 μg/mL (0.5 × MIC). Colonies from multiple GCB + Gen agar plates were replica-plated onto GCB agar plates containing Gen at 8 μg/mL. Gen MICs of identified spontaneous Gen^R^ mutants were subsequently determined as described above.

### Construction of FA19 *fusA2* and FA19 *rpsL fusA2*.

The *fusA* gene was amplified using the primer pair FusA1/FusA2 from the Gen^R^ mutant and subcloned into pBad to create plasmid pBad-*fusA2* Gent^R^. This plasmid was used to transform wild-type FA19, and transformants were selected on GCB agar containing Gen at 16 μg/mL. To give the strains Str^R^, the *rpsL* allele from the previously described FA19 *rpsL* (50) was cloned into pBad and used to transform the FA19 *fusA2* and its parent FA19. *rpsL* transformants were grown on GCB agar containing Str at 200 μg/mL. Strains were confirmed by Sanger sequencing of PCR-amplified *fusA* and *rpsL* gene products.

### Whole-genome sequencing.

WGS of Ng strains was performed with the Illumina MiSeq platform at the UAB Heflin Genomics Core, using paired-end 250-bp reads. To analyze the spontaneous Gen^R^ mutant WGS data, adapter sequences and poor-quality reads were trimmed from raw sequence reads using TrimGalore. Quality-controlled reads were subsequently mapped to the FA19 reference genome (NCBI accession number: CP012026) using Burroughs-Wheeler Alignment tool (BWA) (51). Variants were called using Freebayes (https://arxiv.org/abs/1207.3907) and annotated using SnpEff (52). Common and unique variants were pulled for cross-comparison with VCFtools (53). For assembly and analysis of the *fusA2* mutant genome in the WHO X background, raw WGS reads were assembled using reference-guided *de novo* assembly as described previously (54) with SeqMan NGen version 16.0 (DNASTAR, Madison, WI). In brief, raw reads were mapped against a reference strain (NCBI accession number: NZ_LT592155.1). The mapped reads were then grouped into blocks which were individually *de novo* assembled to form supercontigs. Supercontigs were subsequently scaffolded together using the reference sequences as a guide to aid in gap closure. This method allows for guided *de novo* assembly of the raw reads into complete genomic sequences, allowing comparison of the WHO X Gen^R^ strain to its unique WHO X parent strain. SNPs and indels were called using ArrayStar version 16.0 (DNASTAR, Madison, WI). Variant analysis used a stringency filter with minimum variant percentage at 80%, probability that the base does not match the reference set equal to 90%, and a minimum depth of 20 independent reads to call SNPs. The FASTQ files for the raw reads can be obtained from the Sequence Read Archive under accession number PRJNA801231.

### Determination of protein synthesis.

The assay was performed as previously described by Stubbings et al. (55), with some changes. Briefly, Ng was grown to mid-logarithmic phase in GC broth + 0.5× supplements I and II. Cultures were adjusted to 5 × 10^8^ cells/mL and added to tubes containing 2 μCi of l-[3,4,5-^3^H(N)]-leucine (Perkin Elmer, Waltham, MA) in the presence of Gen or the vehicle control (double-distilled H_2_O), followed by incubation at 37°C in a shaking water bath for 30 min. Ice-cold trichloroacetic acid (TCA, 10% wt/vol) was then added and reactions were allowed to incubate on ice for 1 h to precipitate the protein content. Cultures were pelleted and washed three times with pre-warmed, un-supplemented GC broth with 10% TCA to remove unincorporated tritium. The precipitate was collected on 25-mm GF/C glass microfiber filters (Whatman, Maidstone, United Kingdom) and washed twice with solution containing 10% wt/vol TCA and 1% vol/vol acetic acid. Filters were dried overnight and then counted using CytoScint Liquid Scintillation Cocktail (MP Biomedicals). A non-radioactive experiment was conducted simultaneously to obtain viable counts after exposure to Gen (CFU). Macromolecular synthesis was expressed as disintegrations per minute (dpm) incorporated adjusted by CFU (56). The following equation was used to compare incorporation in the wild-type versus the mutant: [(DPM_mutant@30mins_/CFU_mutant@30mins_)/(DPM_mutant@0mins_/CFU_mutant@0mins_)]/[(DPM_WT@30mins_/CFU_WT@30mins_)/(DPM_WT@0mins_/CFU_WT@0mins_)].

### Analysis of the *fusA* gene in international strains.

All available nucleotide sequences for *fusA* were downloaded from the *Neisseria* PubMLST database as previously described, using the FA19 *fusA* sequence as the reference (39). Strains for which no sequence could be acquired were excluded from analysis. Called nucleotide sequences were aligned to the FA19 sequence using MegAlign version 16.0 (DNASTAR, Madison, WI) and NCBI BLAST (https://blast.ncbi.nlm.nih.gov/Blast.cgi) (57). SNPs were validated by the MegAlign version 16.0 (DNASTAR, Madison, WI) software.

### Site-directed mutagenesis of *fusA*.

To determine if other point mutations in *fusA* could result in Gen resistance, we utilized the Quickchange Lightning Site-Directed Mutagenesis kit (Agilent) to replace the specific residues identified in the international study. Mutations were made in the pCR4-*fusA* plasmid. Primers for site-directed mutagenesis were designed utilizing the Agilent tool (https://www.agilent.com/store/primerDesignProgram.jsp). For allelic replacement of the chromosomal *fusA* region, constructs containing the desired point mutations were transformed into FA19 and selected on GCB agar containing 8, 12, or 16 μg/mL of Gen. For mutations which did not cause a change in the Gen MIC, we used congression transformation of the mutagenic plasmid with the pBad-*rpsL* plasmid and selected them on GCB agar containing Str (200 μg/mL). PCR screening was used for initial identification of transformants. DNA sequencing was used to confirm that, for each strain, the entire *str* locus contained only the desired point mutation(s).

### Competitive infection of the murine lower genital tract.

Competitive infections were conducted as described (58). Briefly, female BALB/c mice (6 to 8 weeks old; Charles River Laboratories Inc., Wilmington, MA; NCI Frederick strain of inbred BALB/cAnNCr mice, strain code 555) were treated with 0.5 mg of Premarin given 2 days prior to, the day of, and 2 days after bacterial inoculation to increase susceptibility to N. gonorrhoeae. Mice were also given antibiotics to suppress the overgrowth of commensal flora that occurs under the influence of estrogen. Groups of mice were inoculated vaginally with similar numbers of FA19 *rpsL* and isogenic FA19 *rpsL fusA2* bacteria (total dose: 10^6^ CFU; 7 mice/group). Vaginal swabs were collected on days 1, 3, and 5 postinoculation and suspended in 100 μL GCB. Swab suspensions and inocula were cultured quantitatively on GCB supplemented with Str (FA19 *rpsL*; total number of CFU) and on GCB with Str and Gen (FA19 *rpsL fusA2*; CFU). Results are expressed as the competitive index (CI), as determined with the following equation: CI = [mutant CFU (output)/wild-type CFU (output)]/[mutant CFU (input)/wild-type CFU (input)]. A detection limit of 1 CFU was assigned for a strain which was not recovered from an infected mouse. A CI of >1 indicates that the mutant is more fit than the WT strain.

### Statistical methods.

All data are expressed as means with standard deviation (SD). Statistical significance between all quantitative data was analyzed by Student’s *t* tests or one-way analysis of variance followed by Tukey’s honestly significant difference *post hoc* test. Statistical significance was set at *P* < 0.05.

### Ethics statement.

Animal experiments were conducted at the Uniformed Services University of the Health Sciences (USUHS) according to the guidelines of the Association for the Assessment and Accreditation of Laboratory Animal Care under protocol no. MIC19-488, approved by the University’s Institutional Animal Care and Use Committee (IACUC). The USUHS animal facilities meet the housing service and surgical standards set forth in the “Guide for the Care and Use of Laboratory Animals,” NIH Publication no. 85-2, and USU Instruction no. 3203, “Use and Care of Laboratory Animals.” Animals were maintained under the supervision of a full-time veterinarian. For all experiments, mice were humanely euthanized by trained personnel upon reaching the study endpoint, using a compressed CO_2_ gas cylinder in the Laboratory of Animal Resources as per Uniformed Services University euthanasia guidelines (IACUC policy 13), which follow those established by the 2013 American Veterinary Medical Association Panel on Euthanasia (https://www.usuhs.edu/mps/facilities-resources).

### Data availability.

The data sets which support the findings of this study are available from the corresponding author upon request.
