# Supplementary material for: A Single Amino Acid Substitution in Elongation Factor G Can Confer Low-Level Gentamicin Resistance in Neisseria gonorrhoeae
Source: Antimicrob Agents Chemother. 2022 Apr 25;66(5):e00251-22. doi: 10.1128/aac.00251-22 (PMC9112995; doi:10.1128/aac.00251-22)
Supplement: Supplemental file 1 — Fig. S1 and S2 and Tables S1 to S5. Download aac.00251-22-s0001.pdf, PDF file, 1.2 MB [file aac.00251-22-s0001.pdf]

Supplementary Information

A single amino acid substitution in elongation factor-G can confer low-level gentamicin resistance in *Neisseria gonorrhoeae*

Concerta L. Holley[1], Vijaya Dhulipala[1], Jacqueline Balthazar[1], Adriana Le Van[2], Afrin A. Begum[2], Shao-Chun Chen[3,4], Timothy D. Read[5,6], Mitch Matoga[7], Irving F. Hoffman[8], Daniel Golparian[9], Magnus Unemo[9,10], Ann E. Jerse[2] and William M. Shafer[1,6,11*]

[1]Department of Microbiology and Immunology Emory University School of Medicine, Atlanta, GA, 30032, USA, [2]Department of Microbiology and Immunology, Uniformed Services University, Bethesda, Maryland, USA, [3]Institute of Dermatology and Hospital for Skin Diseases, Chinese Academy of Medical Sciences and Peking Union Medical College, Nanjing, People's Republic of China, [4]National Center for Sexually Transmitted Diseases Control, Chinese Center for Disease Control and Prevention, Nanjing, People's Republic of China, [5]Department of Medicine and [6]The Emory Antibiotic Resistance Center, Emory University School of Medicine, Atlanta, GA, 30032, USA, [7]UNC Project Malawi, Lilongwe, Malawi, [8]Department of Medicine, University of North Carolina at Chapel Hill, Chapel Hill, NC, USA, [9]WHO Collaborating Centre for Gonorrhoea and Other STIs, National Reference Laboratory for STIs, Department of Laboratory Medicine, Clinical Microbiology, Faculty of Medicine and Health, Örebro University Hospital, Örebro, Sweden, [10]Institute for Global Health, University College London, London, United Kingdom, and [11]Laboratories of Bacterial Pathogenesis, Veterans Affairs Medical Center, Decatur, GA, 30039, USA

CONTENTS

Supplemental Figure S1. EF-G Protein sequence alignment of *N. gonorrhoeae*, *E. coli*, *N. meningitidis*, and *P. aeruginosa*

Supplemental Figure S2. The *fusA2* mutation does not impact *in vitro* growth or macromolecular synthesis in the presence of gentamicin.

Supplemental Table S1. Nucleotide changes observed in the FA19 Gen^R strain compared to the parent FA19 strain as determined by Whole Genome Sequencing

Supplemental Table S2. Nucleotide changes observed in the WHO X *fusA2* strain compared to the parent WHO X strain as determined by Whole Genome Sequencing

Supplemental Table S3. Mutations in the *fusA* gene found through analysis of international PUBMLST data

Supplemental Table S4. Primers used in this study

Supplemental Table S5. Bacterial Strains and plasmids used in this study

Supplemental File References

Supplemental Figure S1.

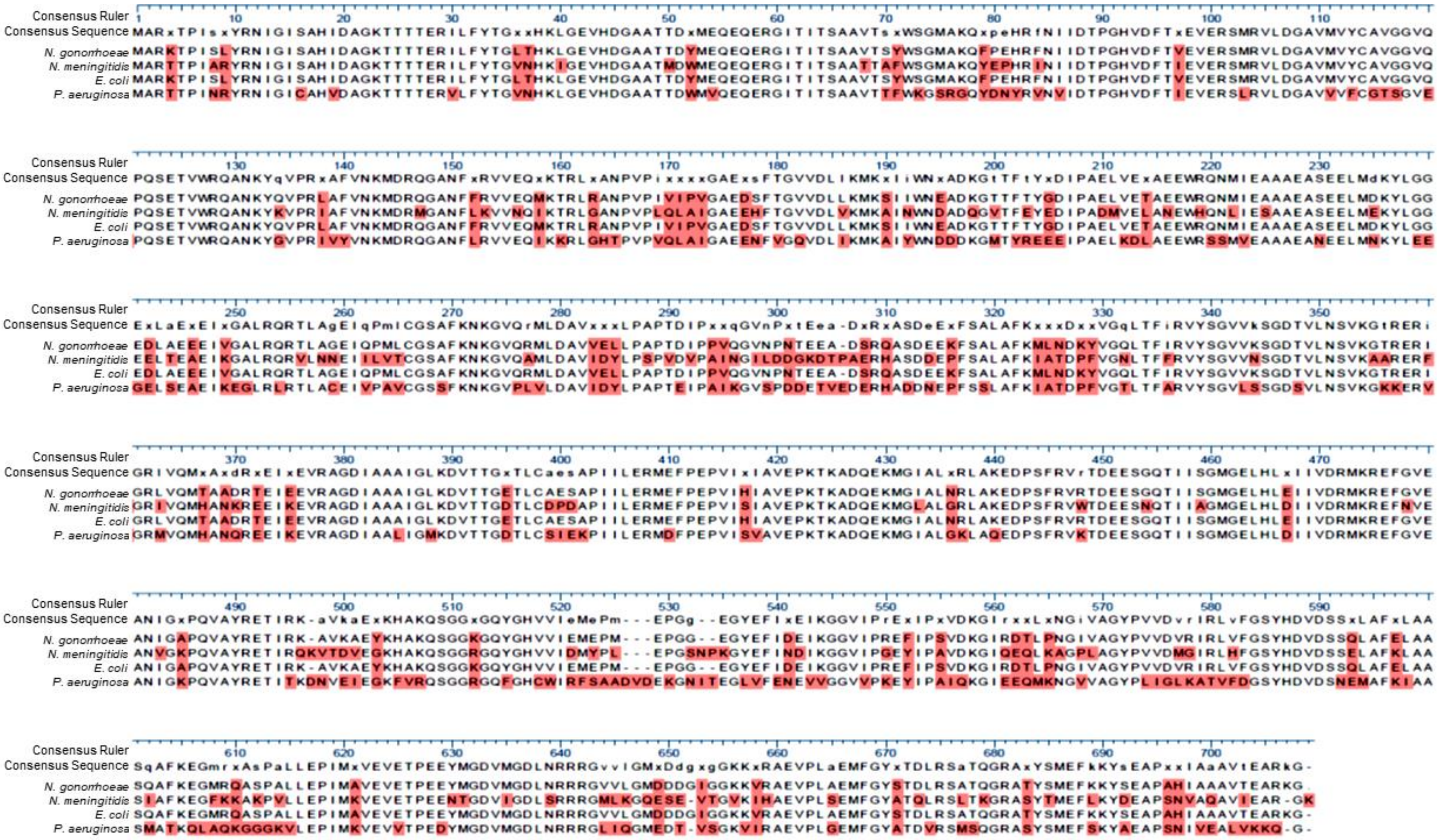

Supplemental Figure S1. Elongation factor G (EF-G) Protein sequence alignment of *N. gonorrhoeae, E. coli, N. meningitidis,* and *P. aeruginosa.* Alignment was performed using MegAlign Version 16.0 (DNASTAR, Madison, WI) and the MUSCLE algorithm. Red highlighting indicates amino acid is different than consensus. If there are two amino acids at a given position, the consensus is called as "x" and is notated as by the software as different from the consensus. The black asterisk indicates location of the A563V in the multi-species alignment. Sequences used in the above comparison: *N. gonorrhoeae* FA19 (Accession #: WP_003690097.1); *E. coli* K-12 substr. MG1655 (Accession #: AAC76365.1); *N. meningitidis* ATCC 13091 (Accession #: EFM03260.1); and *P. aeruginosa* PAO1 (Accession #: NP_252956.1)

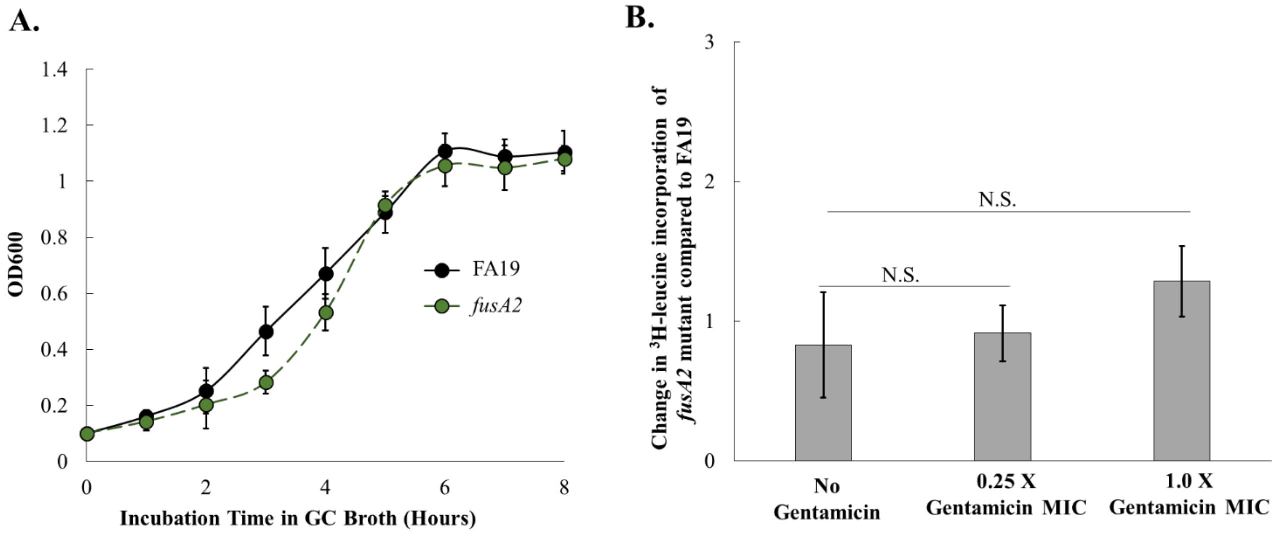

Supplemental Figure S2. Presence of the *fusA2* allele does not impact *in vitro* growth or protein synthesis. A) Growth of FA19 compared to *fusA2* mutant. Average of 4 independent biological assays. **B)** Change in incorporation of radiolabeled precursor [^{3}H-leucine] was quantified in the presence of absence of 0.25X (2 and 8 µg/ml) of 1X (8 and 32 µg/ml) the respective strain MIC of FA19 and the *fusA2* mutant. Each assay represents 4 independent biological assays. Mutant radioactive disintegrations per minute (DPMs) were compared to wild-type DPMs in the presence or absence of gentamicin using the equation: $[(DPM_{mutant@30mins} / CFUs_{mutant@30mins}) / (DPM_{mutant@0mins} / CFUs_{mutant@0mins})] / [(DPM_{WT@30mins} / CFUs_{WT@30mins}) / (DPM_{WT@0mins} / CFUs_{WT@0mins})]$. Statistical analysis was calculated by a one-way ANOVA. P-value of (*$P \leq 0.05$) is considered as statistically significant. N.S., not significant**.**

Supplemental Table S1. Nucleotide changes observed in the FA19 Gen^{R} strain compared to the parent FA19 strain as determined by whole genome sequencing

Intragenic								
**POS**	**FA19**	**Gen^{R}**	**EFFECT**	**IMPACT**	**GENE**	**GENEID**	**Nucleotide Change in Gen^{R}**	**Amino Acid Change in Gen^{R}**
164784	A	T	missense_variant	MODERATE	VT05_00173	VT05_00173	c.26T>A	p.Ile9Lys
201352	T	G	missense_variant	MODERATE	VT05_00219	VT05_00219	c.111A>C	p.Lys37Asn
240533	G	A	missense_variant	MODERATE	VT05_00267	VT05_00267	c.533G>A	p.Arg178Gln
368794	A	T	missense_variant	MODERATE	mboIIM	VT05_00379	c.1740T>A	p.His580Gln
385408	A	C	missense_variant	MODERATE	yhjX	VT05_00393	c.1385A>C	p.Asn462Thr
480228	A	G	missense_variant	MODERATE	dnaX_2	VT05_00485	c.1135T>C	p.Ser379Pro
483738	A	C	missense_variant	MODERATE	VT05_00487	VT05_00487	c.162A>C	p.Lys54Asn
494251	A	G	missense_variant	MODERATE	rpe	VT05_00501	c.92A>G	p.Asp31Gly
541309	G	T	missense_variant	MODERATE	VT05_00548	VT05_00548	c.717G>T	p.Leu239Phe
686931	C	A	missense_variant	MODERATE	VT05_00702	VT05_00702	c.535G>T	p.Asp179Tyr
686948	T	G	missense_variant	MODERATE	VT05_00702	VT05_00702	c.518A>C	p.Lys173Thr
687148	A	C	missense_variant	MODERATE	VT05_00702	VT05_00702	c.318T>G	p.Cys106Trp
693334	T	G	missense_variant	MODERATE	sucB	VT05_00709	c.955A>C	p.Thr319Pro
828689	C	A	missense_variant	MODERATE	VT05_00849	VT05_00849	c.224C>A	p.Pro75His
834948	A	C	missense_variant	MODERATE	VT05_00856	VT05_00856	c.455A>C	p.Tyr152Ser
876024	G	T	missense_variant	MODERATE	VT05_00899	VT05_00899	c.113G>T	p.Arg38Leu
879887	T	G	missense_variant	MODERATE	VT05_00906	VT05_00906	c.137T>G	p.Met46Arg
879893	T	G	missense_variant	MODERATE	VT05_00906	VT05_00906	c.143T>G	p.Leu48Trp
879923	T	G	missense_variant	MODERATE	VT05_00906	VT05_00906	c.173T>G	p.Leu58Trp
879929	C	A	missense_variant	MODERATE	VT05_00906	VT05_00906	c.179C>A	p.Ala60Asp
883110	A	C	stop_lost&splice_region_variant	HIGH	VT05_00912	VT05_00912	c.984A>C	p.Ter328Cysext*?
932311	C	T	missense_variant	MODERATE	trpD	VT05_00965	c.731G>A	p.Gly244Glu
1046459	A	C	non_coding_transcript_exon_variant	MODIFIER	VT05_01068	VT05_01068	n.12T>G	

POS	FA19	Gent^{R}	EFFECT	IMPACT	GENE	GENEID	Nucleotide Change in Gen^{R}	Amino Acid Change in Gen^{R}
1142402	T	G	missense_variant	MODERATE	mafA1_2	VT05_01166	c.299A>C	p.Tyr100Ser
1173466	C	A	missense_variant	MODERATE	ubiB	VT05_01200	c.450G>T	p.Leu150Phe
1205057	A	C	missense_variant	MODERATE	VT05_01227	VT05_01227	c.370A>C	p.Met124Leu
1212779	T	G	missense_variant	MODERATE	VT05_01233	VT05_01233	c.28T>G	p.Leu10Val
1212978	G	T	missense_variant	MODERATE	VT05_01233	VT05_01233	c.227G>T	p.Arg76Leu
1304709	G	A	stop_gained	HIGH	VT05_01321	VT05_01321	c.220C>T	p.Gln74*
1345448	A	C	missense_variant	MODERATE	mafA1_3	VT05_01360	c.272A>C	p.Asn91Thr
1358992	T	G	missense_variant	MODERATE	aroE	VT05_01377	c.149T>G	p.Val50Gly
1363893	T	G	missense_variant	MODERATE	kdsD	VT05_01384	c.475A>C	p.Thr159Pro
1408758	T	G	missense_variant	MODERATE	VT05_01447	VT05_01447	c.842A>C	p.Glu281Ala
1453346	CGCTT(x14) T	CGCTT(x13)T	frameshift_variant	HIGH	VT05_01496	VT05_01496	c.11_14delAAG C	p.Ser22fs
**1618837**	**G**	**A**	**missense_variant**	**MODERATE**	**fusA**	**VT05_01666**	**c.1688C>T**	**p.Ala563Val**
1639576	T	G	missense_variant	MODERATE	VT05_01689	VT05_01689	c.74A>C	p.Tyr25Ser
1641513	G	T	missense_variant	MODERATE	topA	VT05_01692	c.2254C>A	p.Gln752Lys
1849678	CTTT	TTTC	missense_variant	MODERATE	pilE1_3	VT05_01901	c.197_200delAA AGinsGAAA	p.GluArg66GlyLys
2133196	C	A	missense_variant	MODERATE	htrB_2	VT05_02180	c.665C>A	p.Ala222Glu
2138377	A	C	missense_variant	MODERATE	VT05_02183	VT05_02183	c.377A>C	p.Asp126Ala
2138416	A	C	missense_variant	MODERATE	VT05_02183	VT05_02183	c.416A>C	p.Lys139Thr
2175862	A	C	missense_variant	MODERATE	VT05_02223	VT05_02223	c.365A>C	p.Lys122Thr

Intergenic

POS	FA19	Gent^{R}	EFFECT	IMPACT	GENE	GENEID	Nucleotide Change in Gen^{R}	Amino Acid Change in Gen^{R}
302966	G	T	intergenic_region	MODIFIER	VT05_00318-uvrC	VT05_00318-VT05_00319	n.302966G>T	
467865	T	G	intergenic_region	MODIFIER	VT05_00470-VT05_00471	VT05_00470-VT05_00471	n.467865T>G	
580247	C	A	intergenic_region	MODIFIER	tqsA-VT05_00592	VT05_00591-VT05_00592	n.580247C>A	
843536	G	T	intergenic_region	MODIFIER	piiC_3-VT05_00867	VT05_00866-VT05_00867	n.843536G>T	
843569	G	T	intergenic_region	MODIFIER	piiC_3-VT05_00867	VT05_00866-VT05_00867	n.843569G>T	

POS	FA19	$Gent^{R}$	EFFECT	IMPACT	GENE	GENEID	Nucleotide Change in Gen^{R}	Amino Acid Change in Gen^{R}
843600	A	C	intergenic_region	MODIFIER	piiC_3-VT05_00867	VT05_00866-VT05_00867	n.843600A>C	
843642	G	C	intergenic_region	MODIFIER	piiC_3-VT05_00867	VT05_00866-VT05_00867	n.843642G>C	
982706	T	C	intergenic_region	MODIFIER	pgsA-VT05_01014	VT05_01013-VT05_01014	n.982706T>C	
1046476	A	C	intergenic_region	MODIFIER	VT05_01068-coaBC	VT05_01068-VT05_01069	n.1046476A>C	
1144245	C	A	intergenic_region	MODIFIER	lagD_2-VT05_01169	VT05_01168-VT05_01169	n.1144245C>A	
1384732	A	C	intergenic_region	MODIFIER	VT05_01416-VT05_01417	VT05_01416-VT05_01417	n.1384732A>C	
1458426	T	G	intergenic_region	MODIFIER	VT05_01504-comEA_3	VT05_01504-VT05_01505	n.1458426T>G	
2051141	A	C	intergenic_region	MODIFIER	VT05_02101-VT05_02102	VT05_02101-VT05_02102	n.2051141A>C	
2124563	T	G	intergenic_region	MODIFIER	ybeY-hemC	VT05_02170-VT05_02171	n.2124563T>G	
2126014	C	T	intergenic_region	MODIFIER	hemC-VT05_02172	VT05_02171-VT05_02172	n.2126014C>T	

Definitions:
POS = position of the variant or SNPS in the genome relative to the FA19 published sequence (Accession #: CP01202)
EFFECT = How the variant or SNP effects the gene transcript (example: causes a frameshift, missense variant or gains a stop) as defined by SnpEFF
IMPACT = Putative impact of how deleterious the variant is on the gene as predicted by SnpEff (example: High (frameshift or stop gained), Moderate (missense variant), or Low (synonymous variant) impact
GENE = common gene name
GENEID = gene id as defined by the FA19 published sequence

Supplemental Table S2. Nucleotide changes observed in the WHO X *fusA2* strain compared to the parent WHO X strain as determined by whole genome sequencing

Intragenic

Ref Pos	Gene Name	NGO#	protein description	WHO X Variant - Called Seq	WHO X *fusA2* Variant - Called Seq	WHO X Variant - Amino Acid Change	WHO X *fusA2* Variant - Amino Acid Change	WHO X Variant - Classification	WHO X *fusA2* Variant - Classification
21071	NGEG_RS13010 (pseudo)	NGO_1140	T cell/B cell stimulating protein TspB	G>T	GC>TT			Change, genic	Change, genic
72286	NGEG_RS0107345	NGO_r12	16S ribosomal RNA	A	A>G			No change, non-coding RNA	Change, non-coding RNA
24818	NGEG_RS0107030	NGO_1649	TIGR02594 family protein	G	G > del 1		p.A167fs	No change	Frameshift
248302	NGEG_RS0108295	NGO_09965	Opacity protein opA54	G>A	G	p.A145V		Non-synonymous	No change
248313	NGEG_RS0108295	NGO_09965	Opacity protein opA54	GC>CG	G	p.S141T		Non-synonymous	No change
248323	NGEG_RS0108295	NGO_09965	Opacity protein opA54	C>T	C	p.G138E		Non-synonymous	No change
24015	NGEG_RS0107020	NGO_1648	IS110-like element ISNgo3 family transposase	C	C>T		p.R315.	No change	Nonsense
**227097**	**fusA**	**NGO1843**	**elongation factor G**	**G**	**G>A**		**p.A563V**	**No change**	**Non-synonymous**

Intergenic

Ref Pos	Intergenic region		Surrounding protein descriptions	WHO X Variant - Called Seq	WHO X *fusA2* Variant - Called Seq	WHO X Variant - Classification	WHO X *fusA2* Variant - Classification
18290	NGEG_RS0106990	NGEG_RS0106995	tranposase-hypothetical	CGC > del 3	C > del 1	Change, intergenic	Change, intergenic
18294	NGEG_RS0106990	NGEG_RS0106995	tranposase-hypothetical	C>T, - > ins A	C>T	Change, intergenic	Change, intergenic

Ref Pos	Intergenic region		Surrounding protein descriptions	WHO X Variant - Called Seq	WHO X *fusA2*Variant - Called Seq	WHO X Variant - Classification	WHO X *fusA2* Variant - Classification
18295	NGEG_RS0106990	NGEG_RS0106995	tranposase-hypothetical	AA>TT	A>T	Change, intergenic	Change, intergenic
18331	NGEG_RS0106990	NGEG_RS0106995	tranposase-hypothetical	A>G	A	Change, intergenic	No change, intergenic
18354	NGEG_RS0106990	NGEG_RS0106995	tranposase-hypothetical	T>G	T	Change, intergenic	No change, intergenic
25658	NGEG_RS17165	NGEG_RS0107055	hypothetical-hypothetical	T>C	T	Change, intergenic	No change, intergenic
25660	NGEG_RS17165	NGEG_RS0107055	hypothetical-hypothetical	A>G	A	Change, intergenic	No change, intergenic
28060	NGO_1654	NGEG_RS17170	D-tyrosyl-tRNA(Tyr) deacylase--hypothetical	T>G	T	Change, intergenic	No change, intergenic
41775	pilG	glucose-6-phosphate isomerase		TT>CC	T>C	Change, intergenic	Change, intergenic
132750	NGEG_RS14805	NGEG_RS0107630		G>A	G	Change, intergenic	No change, intergenic
183151	NGEG_RS0107895	NGEG_RS0107900		A>C	A	Change, intergenic	No change, intergenic
183158	NGEG_RS0107895	NGEG_RS0107900		T>C	T	Change, intergenic	No change, intergenic
247913	NGEG_RS0108290	NGEG_RS0108295		A>T	AA>TG	Change, intergenic	Change, intergenic
248812	NGEG_RS0108295	NGEG_RS14885		A	- > ins T	No change, intergenic	Change, intergenic
248816	NGEG_RS0108295	NGEG_RS14885		G	G>T	No change, intergenic	Change, intergenic
288445	NGEG_RS0108500	end		A > del 1	A>G	Change, intergenic	Change, intergenic

Definitions:
Ref Pos = position of the variant or SNPS in the genome relative to the WHO X published sequence (Accession #: NZ_LT592155.1)
GENE Name = common gene name
Called Seq = Identity of the nucleotide(s) identified at a given position
Classification = How the variant or SNP effects the gene transcript (example: causes a frameshift, missense variant or gains a stop) as defined by ArrayStar® Version 16.0 (DNASTAR, Madison, WI).

Supplemental Table S3: Mutations in the *fusA* gene found through analysis of international PUBMLST data

Predicted FusA Protein Domain	AA change	nucleotide change	Total # of mutants identified	Continent as defined by PUBMLST database							
				Africa	Antarctica	Asia	Europe	North America	Oceania	South America	Unknown
	R3H	g8a	2				2				
	P6L	c17t	1					1			
	E55K	g160a	25			1	23				1
	S65P	t193c	3				3				
	F84L	t250c	1					1			
	F84L	c252g	1			1					
I	A109V	c326t	1					1			
	T200I	c549t	2				2				
	A234V	c731t	1				1				
	G240S	g718a	1					1			
	E241K	g721a	5			4		1			
	E246K	g736a	3				3				
	A251V	c752t	1				1				
	R277C	c829a	1				1				
	P293L	c878t	1					1			
	A305T	g913a	146	12		4	71	55	1		3
	T347A	a1039g	6				2	4			
	V352I	g1054a	1				1				
II	R356H	c1067a	2			2					
	A367V	c1100t	2				2				
	T371A	a1111g	2					2			
	V376I	g1126a	1				1				
	A382T	g1144a	7				7				

Predicted FusA Protein Domain	AA change	nucleotide change	Total # of mutants identified	Continent as defined by PUBMLST database							
				Africa	Antarctica	Asia	Europe	North America	Oceania	South America	Unknown
III	A418T	g1252a	5			3	1		1		
	A433V	c1298t	1					1			
	E440A	a1319c	1					1			
	S452A	t1354g	1				1				
	E462K	g1384a	1				1				
	V478M	g1432a	1				1				
IV	V497F	g1488t	5						5		
	P522F	c1564t	4				4				
	A563V	c1688t	1			1					
	G564D	g1691a	4				3	1			
	D581N	g1741a	4				4				
	R602H	g1805a	162				145	11	6		
V	A607V	c1820t	1			1					
	V627I	g1879a	1						1		
	R635G	c1903g	1					1			
	R635L	g1904t	1								1
	V651F	g1951t	19			7	9	1	2		
	V651I	g1951a	9				9				
	V651K	g1951a t1952a	1				1				
	E654G	a1961g	1				1				
	P656S	c1965t	1								1
	T665I	c1994t	4			4					
	T674I	c2021t	1								1

Predicted FusA Protein Domain	AA change	nucleotide change	Total # of mutants identified	Continent as defined by PUBMLST database							
				**Africa**	**Antarctica**	**Asia**	**Europe**	**North America**	**Oceania**	**South America**	**Unknown**
**V**	P688S	c2062t	2			2					
	A689V	c2066t	1			1					
	V695L	g2083t	1					1			
	Δ, FS	ATTCTGAA	2				1				1

AA = Amino Acid
Unknown Continent = Originating country of isolate is unknown and a continent cannot be assigned

23

Supplemental Table S4 – Primers used in this study

Primer Name	Sequence (5' to 3')	Purpose
FusA1	GGTGCGTTGAAAAAACGTGAA	Amplification of the *fusA* gene; sequencing of mutants; bioinformatic analysis
FusA2	GGGTGGTTTTACCATGGTCA	Amplification of the *fusA* gene; sequencing of mutants; bioinformatic analysis
FusA3	TGCGGTTCTGCATTTAAAAACA	Amplification of the *fusA* gene; sequencing of mutants
fusA_g913a_F	GACGGCTGTCGGTTTCTTCAGTGTTAGGATTAACA	Site-directed mutagenesis of *fusA* for A305T mutation
fusA_g913a_R	TGTTAATCCTAACACTGAAGAAACCGACAGCCGTC	Site-directed mutagenesis of *fusA* for A305T mutation
fusA_g1805a_F	GGGCAGGAGATGCTTGATGCATACCTTCTTTAAAC	Site-directed mutagenesis of *fusA* for R602H mutation
fusA_g1805a_R	GTTTAAAGAAGGTATGCATCAAGCATCTCCTGCCC	Site-directed mutagenesis of *fusA* for R602H mutation
fusA_g1951t_F	TTCGGCACGGAATTTTTTACCGCCGATACCGTC	Site-directed mutagenesis of *fusA* for V651F mutation
fusA_g1951t_R	GACGGTATCGGCGGTAAAAAATTCCGTGCCGAA	Site-directed mutagenesis of *fusA* for V651F mutation

fusA_c731t_F	CTACGATTTCTTCTTCGACCAGATCTTCACCGCCC	Site-directed mutagenesis of *fusA* for A244V mutation
fusA_c731t_R	GGGCGGTGAAGATCTGGTCGAAGAAGAAATCGTAG	Site-directed mutagenesis of *fusA* for A244V mutation
fusA_g1691a_F	CGTCAACTACAGGATAGTCGGCAACGATACCGTTA	Site-directed mutagenesis of *fusA* for G564D mutation
fusA_g1691a_R	TAACGGTATCGTTGCCGACTATCCTGTAGTTGACG	Site-directed mutagenesis of *fusA* for G564D mutation

Supplemental Table S5 - Bacterial Strains and plasmids used in this study

Strain or plasmid	Genotype or description	Reference or source
N. gonorrhoeae		
FA19	WT strain	(1)
FA19 Gen^{R}	Spontaneous gentamicin resistant strain	This Study
FA19 *fusA2*	Transformant FA19 strain containing a mutation in the *fusA* gene at A563V	This Study
FA19 *rpsL*	WT strain with *rpsL* mutation encoding resistance to Str	(2)
FA19 *rpsL fusA2*	Transformant of FA19 *rpsl* strain containing a mutation in the *fusA* gene at A563V	This Study
WHO X	Ceftriaxone-resistant strain	(3)
WHO X *fusA2*	Transformant of WHO X strain containing a mutation in the *fusA* gene at A563V	This Study
Escherichia coli		
One Shot TOP10	F^{-} *mcr*A Δ(*mrr-hsdRMS-mcrBC*) ϕ80*lacZ*ΔM15 Δ*lacX74 recA1 araD139* (*ara leu*)*7697 galU galK rpsL* (Str^{r}) *endA1 nupG*	Invitrogen (Carlsbad, CA)
XL10-Gold Ultracompetent Cells	TetrΔ(*mcrA*)183 Δ(*mcrCB-hsdSMR-mrr*)*173 endA1 supE44 thi-1 recA1 gyrA96 relA1 lac* Hte [F´ *proAB lacIqZΔM15* Tn*10* (Tetr) Amy Camr]	Agilent Technologies (Santa Clara, CA)
**Plasmids**		
pBAD TOPO TA	Bacterial expression vector for one-step cloning of *Taq*-amplified PCR products	Thermo Fisher Scientific (Waltham, MA)
pBad-*fusA2* Gent^{R}	pBad containing the *fusA* gene from FA19 Gent^{R}	This Study

pCR4 TOPO TA	Bacterial expression vector for one-step cloning of *Taq*-amplified PCR products	Thermo Fisher Scientific (Waltham, MA)
pCR4-*fusA*	pCR4 containing the wild type *fusA* gene for site-directed mutagenesis	This Study
pCR4-*fusA-A305T*	pCR4-*fusA* containing site-directed mutagenesis of A305T	This Study
pCR4-*fusA-R602H*	pCR4-*fusA* containing site-directed mutagenesis of *R602H*	This Study
pCR4-*fusA-A244V*	pCR4-*fusA* containing site-directed mutagenesis of A244V	This Study
pCR4-*fusA-V651F*	pCR4-*fusA* containing site-directed mutagenesis of V651F	This Study
pBad-*rpsl*	pBad containing the streptomycin resistance conferring *rpsl* allele from FA1090	This Study

Supplemental File References

1. Sarubbi FA Jr, Blackman E, Sparling PF. Genetic mapping of linked antibiotic resistance loci in *Neisseria gonorrhoeae*. J Bacteriol. 1974 Dec;120(3):1284-92. PMCID: PMC245913.
2. Jerse AE, Sharma ND, Simms AN, Crow ET, Snyder LA, Shafer WM. A gonococcal efflux pump system enhances bacterial survival in a female mouse model of genital tract infection. Infect Immun. 2003 Oct;71(10):5576-82. PMCID: PMC201053.
3. Ohnishi M, Golparian D, Shimuta K, Saika T, Hoshina S, Iwasaku K, Nakayama S, Kitawaki J, Unemo M. Is *Neisseria gonorrhoeae* initiating a future era of untreatable gonorrhea?: detailed characterization of the first strain with high-level resistance to ceftriaxone. Antimicrob Agents Chemother. 2011 Jul;55(7):3538-45. PMCID: PMC3122416.
